# The effect of follicle size and homogeneity of follicular development on the morphokinetics of human embryos

**DOI:** 10.1007/s10815-017-0935-1

**Published:** 2017-05-04

**Authors:** Semra Kahraman, Caroline Pirkevi Cetinkaya, Murat Cetinkaya, Hakan Yelke, Yesim Kumtepe Colakoglu, Melih Aygun, Markus Montag

**Affiliations:** 1 Assisted Reproductive Technologies and Reproductive Genetics Centre, Istanbul Memorial Hospital, Piyale Pasa Bulvari, Okmeydani, 34385 Istanbul, Turkey; 2 ilabcomm GmbH, Eisenachstr. 34, 53757 Sankt Augustin, Germany

**Keywords:** Follicle size, Follicular development, Homogeneity, Morphokinetics, Blastocyst

## Abstract

**Purpose:**

Our aim was to investigate follicular size (large, ≥17 mm and small, <17 mm) at the time of OPU and homogeneity of follicular development (homogenous development: follicles being present in a homogenous spread of all sizes; heterogeneous: a predominance of small and large follicles) by analysing the morphokinetics of embryo development.

**Methods:**

In this prospective cohort study, 2526 COCs belonging to 187 patients were cultured to day 5. Embryos were evaluated morphokinetically. Four subgroups were defined: large follicles from heterogeneous cycles (LHet) and homogenous cycles (LHom) and small follicles from heterogeneous cycles (SHet) and homogenous cycles (SHom).

**Results:**

Rates of fertilization, blastocyst formation and top and good quality blastocysts were found to be significantly higher in embryos from the LHom group (*p* < 0.001; *p* < 0.001; *p* < 0.001). Small follicles from both homogenous and heterogeneous cycles had significantly lower blastocyst formation and top and good quality blastocyst rates (*p* < 0.001; *p* < 0.001). Embryos from SHet had significantly more direct cleavages (*p* = 0.011). Time to reach blastocyst was shorter in SHom than LHet and LHom (*p* = 0.002; *p* = 0.027, respectively). However, once the blastocyst stage was achieved, implantation rates were not significantly different between subgroups, the highest rate being observed in the LHom group. Multivariable analysis revealed that homogeneity of follicular development and follicular size had a significant effect on blastocyst development and quality (*p* = 0.049; *p* < 0.001, respectively).

**Conclusion:**

Follicular dynamics, illustrated by follicular size and homogeneity of follicular development, influence early human embryo development. Patterns of follicular growth have an impact on embryo quality and viability which is reflected in morphokinetic variables.

**Electronic supplementary material:**

The online version of this article (doi:10.1007/s10815-017-0935-1) contains supplementary material, which is available to authorized users.

## Introduction

This study was undertaken as a result of clinical practitioner observations in an assisted reproductive technology (ART) setting which led to questions regarding a possible relationship between follicular size, homogeneity of follicular development and reaching blastocyst stage and clinical outcomes. To the best of our knowledge, these questions had not so far been studied.

Our study aimed to investigate the clinical relevance of two common controlled ovarian stimulation (COS) parameters: follicular size (large and small) at the time of oocyte pickup (OPU) and homogeneity of follicular development (homogenous development: follicles being present in a homogenous spread of all sizes; heterogeneous: a predominance of small and large follicles) by analysing the morphokinetics of embryo development and to evaluate clinical outcomes.

The reason why some oocytes have better developmental capacity than the other oocytes developed in the same cohort is not known yet. The mechanism underlying the individual response of antral follicles to exogenous gonadotropin has not yet been clearly determined. However, it is known that early antral follicles do not necessarily grow coordinately in response to exogenous gonadotropins to reach simultaneous functional and morphologic maturation, and not necessarily all FSH responding follicles yet have enough LH receptors to respond to the maturation signal introduced by hCG [1, 2]. Moreover, in the GnRH antagonist cycles, a physiological increase in the FSH level during the luteal-follicular transition phase provokes a heterogeneous follicular development leading to a slightly lower maturation rate when compared to agonist cycles. During the early follicular phase, early antral follicles present noticeable size heterogeneities that may be amplified during COS [3]. Thus, multifollicular growth may result in heterogeneous size of follicles, variable growth rate and also may cause secondary and tertiary cohorts [4–7].

There are studies in the literature regarding embryo development and follicular size indicating that better oocytes are obtained from large follicles [8–13]. However, Nivet et al., when studying the impact of follicular size on oocyte quality as measured by embryonic development, demonstrated that medium size follicles yield a better percentage of transferable embryos [14].

Oocyte size is a key factor for meiotic competence while developmental competence is not present before the full size is reached. Intrinsic oocyte quality determines the oocyte’s ability to overcome meiotic arrest and progress to the metaphase II (MII) stage (meiotic competence) and to undergo fertilization and support early embryonic stages (developmental competence) [15]. Nuclear and cytoplasmic maturation are characterized by an increased oocyte diameter, chromatin condensation, transcriptional quiescence and also mitochondrial aggregation towards the nucleus [16, 17]. Therefore, MII oocytes may, because of poor cytoplasmic maturity, result in suboptimal rates of embryo development.

The development of incubators with built-in time-lapse technology has enabled continuous non-invasive monitoring of embryo development from fertilization to blastocyst stage and the possibility of appraising the precise timing of embryonic cell divisions [18–22]. Many studies have tried to connect, more or less conclusively, embryo development, timing of mitotic divisions, embryo viability and implantation. However, time points indicating precise embryo cleavages were shown to be affected by extrinsic factors such as ovarian stimulation protocols, culture conditions, fertilization method and also by patient-specific factors [23–28].

To the best of our knowledge, no previous studies have investigated both follicular size and homogeneity of follicular development by analysing the morphokinetics of embryo development.

## Materials and methods

### Patients

This prospective cohort study was registered at clinicaltrials.gov (NCT02230449) and obtained an ethical approval from the institutional review board (23/06/2014-19). It was conducted in a private IVF clinic between July 2014 and September 2015. The analysis was based on a total of 2526 cumulus oocyte complexes (COCs) belonging to 187 patients with culture until day 5 since 13 patients were excluded (one premature ovulation, one oocyte maturation defect, two low fertilizations, three fertilization failures, two cleavage-stage developmental arrests, four OHSS/freeze-all). In the cases of low fertilization, one patient had only one fertilized oocyte which was abnormal (three pronuclei (PN)) and in the other case, despite more than eight COCs being retrieved, there was only one MII oocyte which presented only one PN and did not cleave. In the two cases of cleavage-stage arrests at the two-cell stage, the blastomeres were significantly different in size, possibly indicating abnormality. The patients included in this study presented various infertility causes and the mean female age was 31.1 years. All protocols were approved by the institutional review board and all patients gave their informed consent prior to their inclusion in the study. Patients were selected based on inclusion criteria (age ≤39 years, body mass index (BMI) <30 kg/m^2^, ≥8 COCs retrieved, <2 previous treatment cycles, hCG trigger) and exclusion criteria (recurrent pregnancy loss, severe endometriosis, PGD or PGS, COC > 24, embryo transfer (ET) < day 5, PCOS, uterine anomaly, severe sperm morphological abnormality such as dominantly macrocephalic or globozoospermic sample or cryptozoospermia, ≤1million motile sperm cells in total ejaculate). All embryos were obtained after fertilization by intracytoplasmic sperm injection (ICSI) and were part of our standard ART program. Embryo development was recorded using time-lapse technology (EmbryoScope™ time-lapse system, Vitrolife, Göteborg, Sweden).

### Ovarian stimulation

The baseline estradiol, LH and progesterone levels were evaluated on cycle day 2, and baseline ultrasound scans were performed on the same day. Depending on the BMI, anti-mullerian hormone (AMH) level, basal antral follicle count and the history, if any, of a previous response to gonadotropins, recombinant FSH (rFSH; Gonal-F®; Merck Serono, Switzerland) was used for ovarian stimulation at a dosage of 150 to 225 IU. The standard dose was 150 IU, when necessary however, depending on body mass index, 225 IU was started. From the fourth or fifth day of rFSH therapy onwards, patients were monitored daily or every other day for hormone levels (estradiol, LH and progesterone whenever needed) and follicular measurements. A daily administration of 0.25 mg GnRH antagonist (Cetrotide®; Merck Serono, Switzerland) was administered when the size of the follicle was at least 12 mm, but never exceeding 13 mm. Follicular maturation was achieved by using 250 μg recombinant hCG (Ovitrelle®; Merck Serono, Switzerland) when at least three follicles reached a minimum mean diameter of at least 17 mm. Transvaginal ultrasound-guided oocyte retrieval was scheduled for 36 h later.

### Follicular size

Each follicular aspiration was performed by the same doctor in order to reduce any possible inaccuracy of measurement to a minimum. Follicles <17 mm at the time of OPU were classified as small while those ≥17 mm were classified as large. Oocytes coming from large and small follicles were incubated separately.

### Homogeneity of multifollicular growth

The data was also evaluated according to the homogeneity of follicular development. Follicles being present in a homogenous spread of all sizes from large (>20 mm) to intermediate (17-20 mm) to small (<17 mm) was considered to be homogenous development, whereas a predominance of large (>20 mm) and small (<17 mm) follicles was considered to be heterogeneous. Each case met the criteria for trigger of a minimum of three follicles of at least 17 mm. Four subgroups have been defined according to the follicular size and cycle homogeneity: small follicles/heterogeneous follicular development (SHet), small follicles/homogenous follicular development (SHom), large follicles/heterogeneous follicular development (LHet) and large follicles/homogenous follicular development (LHom).

### Oocyte retrieval, denudation and ICSI

On the day of OPU, follicles were aspirated separately and COCs were washed in human tubal fluid medium (HTF; Life Global®, Brussels, Belgium). The gynaecologist who performed the pickup informed the embryology laboratory of the size of follicle (large ≥17 mm /small <17 mm) from which each oocyte was derived. One embryologist retrieved oocytes with a second embryologist assisting to ensure smooth operation and to document the process of isolation, identification and positioning of COCs in the culture dish.

The COCs were then incubated for 3.5 h at 6% CO_2_, 5% O_2_ and 37 °C before denudation, which was carried out by mechanical pipetting in ICSI Cumulase® (Origio, Måløv, Denmark). Each COC was denuded separately and the maturation status was then determined. Next, oocytes were allowed to incubate for an additional 30 min. ICSI was then performed in an HTF medium with HEPES (Life Global®, Brussels, Belgium) at ×400 magnification using Olympus IX70 and Olympus IX71 inverted microscopes.

### Embryo culture and incubation

Each of the 12 individual wells of the EmbryoSlide® culture dish was filled with 25 μl of a single step culture medium (Life Global®, Brussels, Belgium), supplemented with 10% Plasmanate (Life Global®, Brussels, Belgium), and all wells were covered with an overlay of 1.5 mL paraffin oil (Life Global®, Brussels, Belgium). Following ICSI, injected oocytes were positioned in the wells of the slide, which was placed in a time-lapse incubator (EmbryoScope™) at 6% CO_2_, 5% O_2_ and 37 °C for 5 days until embryo transfer. The culture medium was refreshed on the afternoon of day 3 by replacing the incubated slide with a new pre-equilibrated slide prepared as described above. Image stacks were acquired at seven focal planes every 15 min, and data were continuously transferred to an external computer, EmbryoViewer® workstation (Vitrolife, Göteborg, Sweden). Embryo development was annotated by one investigator and cross-checked by two other assessors.

### Time-lapse evaluation and embryo scoring

Morphokinetic variables for all cleavage events up to the expanded blastocyst stage were annotated. All relevant events (fertilization, cleavages, morula and blastocyst formation) were checked on a daily basis, and time of cleavage to two-cell embryo (t2) and subsequent divisions t3, t4, t5, t6, t7, t8 and t9+ were recorded in the EmbryoViewer® workstation. The time of all mitotic events was expressed as hours post-ICSI. In order to minimize the variation of ICSI time within oocytes of one patient, ICSI was split between two embryologists above ten oocytes. Therefore, the maximum ICSI duration did not exceed 15 min, which is below the default time interval of each picture taken by the camera of the EmbryoScope™ system. tM was annotated at the end of the compaction process, when compaction was observed to be full with no apparent cell contours. tSB marks the initiation or start of blastulation, the first frame when initiation of a cavity formation is observed. tB indicates a blastocyst, where the ICM and the cavity are formed. tEB shows an expanded blastocyst with 50% thinning of the zona pellucida. Blastocysts were scored according to Gardner’s classification (114–120 h post-ICSI) and selected for transfer based on the final morphology and the score obtained from the morphokinetic ratios published by Çetinkaya and colleagues (CS2–8 = ((t3-t2) + (t5-t4)) / (t8-t2); CS4–8 = (t8-t5) / (t8-t4)) [29].

### Embryo transfer

After embryo transfer, for luteal phase support, patients received a twice daily dose of progesterone gel administered intravaginally (Crinone® 8%; Merck Serono, Switzerland). When pregnancy occurred, a daily dose was continued until the 10th week of gestation. Fourteen days after pickup, serum β-hCG was measured. At 7 weeks, a transvaginal ultrasound was performed to monitor early pregnancy. The implantation rate was calculated by dividing the number of implanted embryos by the total number of transferred embryos.

### Power calculation

An earlier pilot study revealed that top and good quality blastocyst rates for large and small follicles were 45 and 32%, respectively. A power analysis indicated that, for an alpha level of 0.05 and a beta level of 0.20 (power = 0.80), 438 fertilized oocytes were sufficient to detect a significant difference between follicle size groups.

### Statistical analysis

Demographics of patients were reported as minimum, maximum, mean ± sd and 95% confidence interval of the mean. Due to the dependent nature of the data, generalized linear mixed models with logit link function were conducted to analyse the differences of binary variables between four groups, namely LHom, LHet, SHom and SHet. Generalized linear mixed models with linear link function were conducted to analyse the differences of continuous variables between the four subgroups mentioned above.

Generalized linear mixed models with logit link function were conducted to analyse the possible effects of factors on the rate of top and good quality blastocysts. First, five models were conducted separately to test the effects of age, AMH, BMI, homogeneity and follicle size on the rate of top and good quality blastocysts. Second, a multivariable model was conducted, where variables with *p* < 0.20 significance level at univariable analysis were introduced as independent variables. A *p* value of <0.05 was considered statistically significant. All statistical analyses were performed using the MedCalc Statistical Software version 13.2.0 (MedCalc Software bvba, Ostend, Belgium) and R version 3.3.2.

## Results

### Cycle outcomes in the study cohort

A total of 187 patients were prospectively involved in the analysis, using strict inclusion criteria, which allowed the study of a homogeneous, young, infertile patient population with a low BMI and a good ovarian reserve (mean female age 31.1 ± 4.1, AMH 3.1 ± 2.0 ng/mL and BMI 23.6 ± 3.0 kg/m^2^). An average of 14 COCs was collected, 10.1 of which were at MII stage (maturation rate 73.6%). The fertilization rate after ICSI was 76.6%. The fertilized oocytes (71.7%) became grade 1 or 2 embryos on day 3 and 37.2% top or good quality blastocysts on day 5 (Table 1). The clinical and ongoing pregnancy rates achieved in the study cohort were 63.9 and 55.2%, respectively. The early clinical miscarriage rate was 13.7%. Two thirds of patients had surplus frozen blastocysts (66.3%). Finally, a live birth rate of 52% was reached (Supplementary Table 1).

**Table 1:** Patient and cycle characteristics

Patients (*n* = 187)	Min-max	Mean ± sd	95% CI for mean
Female age	18–38	31.13 ± 4.17	30.53 to 31.73
AMH (ng/mL)	0.17–10.70	3.11 ± 2.03	2.82 to 3.41
BMI (kg/m^2^)	16.30–29.70	23.67 ± 3.03	23.23 to 23.11
Follicles (<17 mm at OPU) (%)	8.33–88.89	54.02 ± 17.59	51.48 to 56.56
Follicles (≥17 mm at OPU) (%)	11.11–91.67	45.98 ± 17.59	43.44 to 48.51
COC	5–24	14.03 ± 4.88	13.33 to 14.74
MII	4–24	10.12 ± 3.88	9.56 to 10.68
Maturation rate (%)	31.82–100	73.69 ± 16.88	71.26 to 76.13
Fertilization rate (%)	18.75–100	76.61 ± 14.76	73.81 to 79.65
D3 grade 1 and 2^a^/PN^b^ (%)	0–100	71.70 ± 22.08	68.52 to 74.89
Blastocyst/PN (%)	16.67–100	53.26 ± 17.40	50.75 to 55.77
TQ^c^ and GQ^d^ blastocysts/PN (%)	0–100	37.22 ± 19.10	34.46 to 39.97

*AMH* anti-mullerian hormone, *BMI* body mass index, *COC* cumulus oocyte complex, *MII* metaphase II oocyte

^a^Day 3 grades 1 and 2 embryos have at least six cells, less than 10% fragmentation with even blastomeres or minor unevenness between blastomeres

^b^Fertilized oocyte (PN)

^c^Top quality (TQ) blastocysts are those graded as 3AA, 4AA and 5AA

^d^Good quality (GQ) blastocysts are those graded as 3/4/5BB, AB, BA and 2AA

### Outcomes according to follicular size and homogeneity of follicular development

Maturation and fertilization rates of oocytes deriving from large follicles were significantly higher than those deriving from small follicles. When subcategorizing the data, large follicles developed in homogenous cycles (LHom) had better outcomes than large follicles developed in heterogeneous cycles (LHet), small follicles in homogenous cycles (SHom) and finally small follicles in heterogeneous cycles (SHet). Also, rates of grade 1 and 2 embryos on day 3, blastocyst formation, top and good quality blastocysts were gradually higher in the same order mentioned above.

### Morphokinetic analysis of follicular size and homogeneity of follicular development

**Fig. 1:**
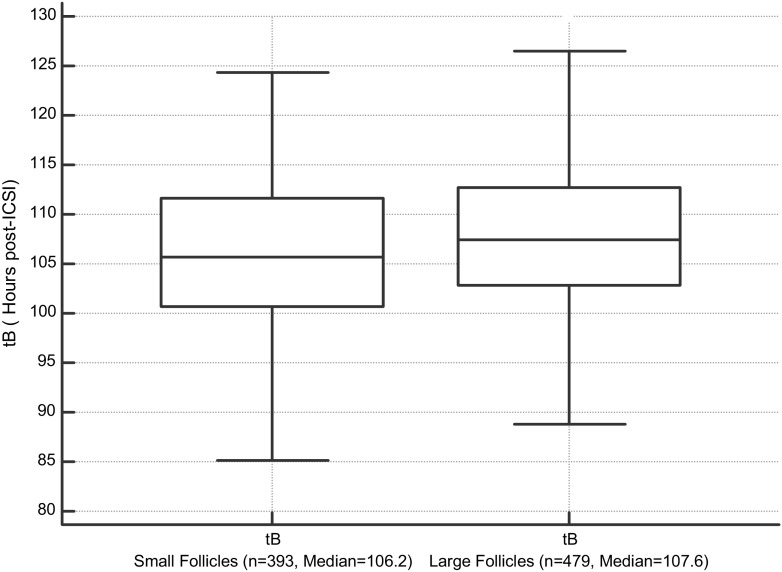
Time to achieve a blastocyst (*tB*) in small vs. large follicles (*tB* is in hours post-ICSI) (*p* = 0.0036)

The kinetics of embryos derived from small and large follicles was evaluated according to cycle homogeneity. Statistically significant differences were found beginning from the first cleavage (t2) until the expanded blastocyst time (tEB) except for t3 and t4. Embryos from small follicles developed faster than embryos originating from large follicles, for all cleavage timings. Embryos from SHet developed faster, except for t9 and tB (Table 2). tB time was significantly different between follicular size and homogeneity groups (*p* = 0.001). Bonferroni corrected post-hoc analysis revealed that tB time was shorter in the SHom group than LHet or LHom groups (*p* = 0.002; *p* = 0.027, respectively), whereas embryos developing from the SHet group had a tB shorter than embryos coming from the LHet group (*p* = 0.002). When median values were compared, the time to achieve a blastocyst for a small follicle was 1.4 h faster than for a large follicle (*p* = 0.0036) (Fig. 1).

**Table 2:** Comparison of outcomes, time intervals and time ratios between groups

(*n* = 2526)	SHet	SHom	LHet	LHom	*p*	Post hoc
Maturation, *n* (%)	409 (63.0)	501 (72.6)	463 (83.7)	535 (84.4)	<0.001*	Shet < Shom< Lhet, Lhom
Fertilization, *n* (%)	321 (78.5)	380 (75.8)	362 (78.2)	469 (87.7)	<0.001*	Shet, Shom, Lhet < Lhom
Day 3 grades 1 and 2, *n* (%)	239 (66.2)	334 (77.0)	314 (78.1)	408 (80.6)	<0.001*	Shet < Shom, Lhet, Lhom
Blastocyst, *n* (%)	234 (36.1)	298 (43.2)	288 (52.1)	397 (62.6)	<0.001*	Shet, Shom < Lhet < Lhom
TQ and GQ, *n* (%)	130 (20.0)	160 (23.2)	159 (28.8)	233 (36.8)	<0.001*	Shet, Shom < Lhet < Lhom
Implantation, *n* (%)	19 (35.2)	19 (47.5)	18 (36.7)	36 (58.1)	0.237	–
Direct cleavage, *n* (%)	93 (26.7)	92 (22.3)	70 (18.2)	82 (16.8)	0.011*	Shet > Lhet, Lhom
t2, mean ± sd	28.32 ± 5.88	27.81 ± 4.99	27.83 ± 5.11	27.38 ± 4.28	0.081	–
t3, mean ± sd	37.15 ± 6.44	37.11 ± 6.00	37.79 ± 6.19	37.66 ± 6.51	0.567	–
t4, mean ± sd	39.90 ± 7.17	39.74 ± 6.94	40.21 ± 6.65	39.51 ± 7.08	0.553	–
t5, mean ± sd	48.27 ± 9.60	48.58 ± 8.87	50.16 ± 8.65	50.10 ± 9.89	0.015*	Shet < Lhet and Shom < Lhom
t6, mean ± sd	52.15 ± 10.11	52.04 ± 8.75	53.88 ± 8.64	53.43 ± 8.87	0.009*	Shet, Shom < Lhet and Shom < Lhom
t7, mean ± sd	55.06 ± 10.52	55.28 ± 9.59	57.26 ± 9.11	56.26 ± 9.75	0.044*	Shet, Shom < Lhet
t8, mean ± sd	57.95 ± 10.75	58.49 ± 10.14	60.42 ± 9.39	59.55 ± 10.55	0.032*	Shet < Lhet
t9, mean ± sd	66.88 ± 11.77	67.04 ± 11.02	69.26 ± 10.88	69.29 ± 11.01	0.008*	Shom < Lhom
tM, mean ± sd	88.21 ± 10.10	88.56 ± 9.36	91.47 ± 10.23	88.91 ± 10.19	<0.001*	Shet, Shom, Lhom < Lhet
tSB, mean ± sd	98.92 ± 8.11	98.57 ± 8.36	101.58 ± 7.98	99.64 ± 8.23	<0.001	Shet, Shom < Lhet and Shom < Lhom
tB, mean ± sd	106.06 ± 7.33	106.30 ± 6.70	108.52 ± 6.52	107.08 ± 7.12	0.001*	Shom < Lhet, Lhom and Shet < Lhet
tEB, mean ± sd	109.92 ± 5.88	111.60 ± 5.45	112.88 ± 4.94	111.88 ± 5.74	<0.001*	Shet < Lhet, Lhom and Shom < Lhet
cc2 (t3-t2), mean ± sd	8.83 ± 5.08	9.36 ± 4.74	10.14 ± 4.90	10.36 ± 5.45	<0.001*	Shet < Lhet, Lhom and Shom < Lhom
s2 (t4-t3), mean ± sd	2.92 ± 4.98	2.58 ± 4.50	2.49 ± 4.78	1.89 ± 3.59	0.010*	Shet, Shom, Lhet > Lhom
cc3 (t5-t3), mean ± sd	11.36 ± 6.83	11.56 ± 6.19	12.60 ± 6.23	12.51 ± 6.92	0.016*	Shet < Lhet, Lhom and Shom < Lhom
t5-t4, mean ± sd	8.59 ± 6.56	9.01 ± 6.23	10.30 ± 6.41	10.62 ± 6.57	<0.001*	Shet, Shom < Lhet, Lhom
t8-t5, mean ± sd	10.12 ± 8.47	10.46 ± 8.58	10.44 ± 8.69	9.64 ± 8.37	0.526	–
t8-t2, mean ± sd	30.42 ± 9.23	31.37 ± 8.70	33.14 ± 8.16	32.51 ± 9.32	0.001*	Shet, Shom < Lhet, Lhom
CS2-8, mean ± sd	0.59 ± 0.30	0.61 ± 0.28	0.63 ± 0.28	0.67 ± 0.26	0.001*	Shet < Lhet, Lhom and Shom < Lhom
CS4-8, mean ± sd	0.52 ± 0.34	0.51 ± 0.34	0.48 ± 0.32	0.44 ± 0.31	0.002*	Shet, Shom > Lhom

Generalized linear mixed models were conducted for all comparisons. Bonferroni corrected post-hoc results were reported. Time of cleavage to two-cell embryo (t2), and subsequent divisions t3, t4, t5, t6, t7, t8 and t9+, were annotated. tM, tB and tEB are times to achieve a morula, a blastocyst and an expanded blastocyst, respectively. CS2–8 = ((t3-t2) + (t5-t4)) / (t8-t2); CS4–8 = (t8-t5) / (t8-t4) [28]

*tSB* time for start of blastulation

*p < 0.05

Another difference observed, when embryos developing from small and large follicles in homogenous or heterogeneous cycles were compared, was the significantly different rate of direct cleavages (t3-t2 < 5 h) (*p* = 0.011) (Table 2). Bonferroni corrected post-hoc analysis revealed that the direct cleavage rate was higher in the SHet group (26.7%) than in the LHet (18.2%) (*p* = 0.016) and in the LHom groups (16.8%) (*p* = 0.006).

Also, when time intervals and time ratios were evaluated, cc2 (t3-t2), s2 (t4-t3), cc3 (t5-t3), t5-t4, t8-t2, CS2–8 ([(t3-t2) + (t5-t4)] / (t8-t2)) and CS4–8 ((t8-t5) / (t8-t4)) were significantly different between follicular size and homogeneity of follicular development subgroups (Table 2). cc2 and cc3 time intervals were significantly different between all subgroups (*p* < 0.001 and *p* = 0.016, respectively). Bonferroni corrected post-hoc analyses revealed that cc2 and cc3 time intervals were shorter in embryos developing from the SHet group than in embryos developing from LHet or LHom groups (for cc2, *p* = 0.003; *p* = 0.001; and for cc3, *p* = 0.020; *p* = 0.039, respectively). When looking at homogenous cycles, cc2 and cc3 were shorter in embryos developing from small follicles than in those from large follicles (*p* = 0.004; *p* = 0.026; *p* = 0.002, respectively). Also, CS2–8 time interval showed a similar pattern in the aforementioned four categories (*p* = 0.045; *p* = 0.002; *p* = 0.002, respectively).

### Embryo developmental arrests according to follicular size

**Fig. 2:**
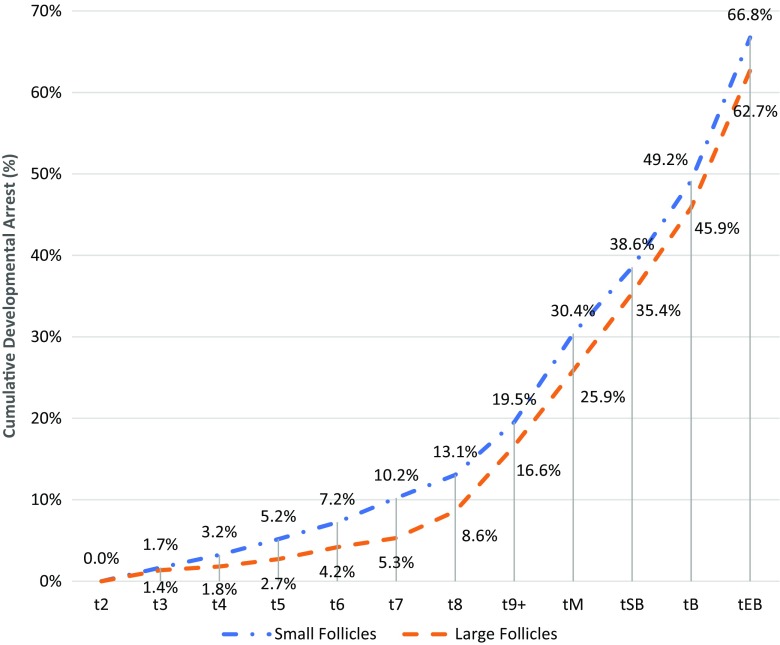
Cumulative developmental arrest (%) of embryos originating from small and large follicles from t2 to tEB

Embryos originating from small follicles had a higher arrest rate than embryos originating from large follicles when analysing those having a first cleavage t2 (Fig. 2).

Cumulative developmental arrest rates of embryos originating from small and large follicles were calculated by adding all arrested embryos up to that time point for each category divided by embryos having achieved the two-cell stage. When looking at the time to achieve the eight-cell stage (t8), 91.4% of embryos developing from large follicles reached t8, compared to only 86.9% of embryos developing from small follicles with a 4.5% lower arrest rate in the former group (*p* = 0.0032) (Fig. 2). No significant differences were found between the developmental arrest rates of embryos from t8 to expanded blastocyst (tEB).

### Implantation rates according to follicular size and homogeneity of follicular development

Although not statistically significant, embryos developing from the LHom group had a higher implantation rate when compared first to SHom, second to LHet and finally to SHet groups (58.1, 47.5, 36.7, 35.2%, respectively; *p* = 0.237) (Table 2).

### Univariable and multivariable analyses of factors affecting blastocyst quality

Univariable analyses revealed that AMH, homogeneity of follicular development and follicular size had a significant effect on blastocyst quality (*p* = 0.010; *p* = 0.018; *p* < 0.001, respectively). One unit increase in AMH level resulted in a 1.086-fold increase in top or good quality blastocysts development [OR (95% CI) 1.086 (1.020, 1.157); *p* = 0.010]. For follicles derived from homogenous cycles, the odds of resulting in top or good quality blastocysts were 1.370-fold larger than the odds for heterogeneous follicles becoming top or good quality blastocysts [OR (95% CI) 1.370 (1.055, 1.780); *p* = 0.010]. For large follicles, the odds of developing into top or good quality blastocysts were 1.803-fold larger than the odds for small follicles resulting in top or good quality blastocysts [OR (95% CI) 1.803 (1.494, 2.177); *p* < 0.001] (Table 3).

**Table 3:** Univariable and multivariable analyses of factors affecting blastocyst quality

	Univariable			Multivariable		
	*t*	*p*	Exp(*β*) (95% CI)	*t*	*p*	Exp(*β*) (95% CI)
Female age	−1.400	0.162	0.978 (0.949, 1.009)	−1.003	0.316	0.984 (0.955, 1.015)
AMH	2.572	0.010*	1.086 (1.020, 1.157)	2.231	0.026*	1.075 (1.009, 1.146)
BMI	0.799	0.424	1.018 (0.975, 1.063)	–	–	–
Homogenous cycles	2.364	0.018*	1.370 (1.055, 1.780)	1.970	0.049*	1.302 (1.001, 1.693)
Follicle size (≥17 mm at OPU)	6.148	<0.001*	1.803 (1.494, 2.177)	6.101	<0.001*	1.797 (1.488, 2.169)

Generalized linear mixed models were conducted for univariable and multivariable analyses

*p < 0.05

Multivariable analysis revealed that AMH, homogeneity of follicular development and follicular size had a significant effect on blastocyst quality (*p* = 0.010; *p* = 0.018; *p* < 0.001, respectively). One unit increase in AMH level resulted in a 1.075-fold increase in top or good quality blastocysts development [OR (95% CI) 1.075 (1.009, 1.146); *p* = 0.026]. For homogenous cycles, the odds of becoming top or good quality blastocysts were 1.302-fold larger than the odds for heterogeneous follicles developing into top or good quality blastocysts [OR (95% CI) 1.302 (1.001, 1.693); *p* = 0.049]. For large follicles, the odds of resulting in top or good quality blastocysts were 1.797-fold larger than the odds for small follicles becoming top or good quality blastocysts [OR (95% CI) 1.797 (1.488, 2.169); *p* < 0.001] (Table 3).

## Discussion

To the best of our knowledge, this study describes for the first time the impact of follicular dynamics, illustrated by follicular size and homogeneity of follicular development, on early human embryo development. One of the main findings emerging from this study is that a significantly higher number of good and top quality blastocysts suitable for transfer and freezing were observed in the LHom group. Although not significantly different between the subgroups, the highest implantation rate was also observed in the LHom group.

Embryos originating from the LHom group outperformed significantly those from the SHom group in terms of maturation, fertilization, blastocyst formation and top and good quality blastocyst rates. Also, embryos originating from the SHet group showed significantly higher rates of developmental arrest or abnormal morphokinetic patterns such as direct cleavages, when compared to large follicles. However, once an embryo originating from a small follicle achieved the eight-cell stage, no difference in the developmental progression up to the blastocyst stage was observed when compared to embryos originating from large follicles. In order to control for potential confounders, a multivariate analysis was conducted, revealing that in addition to AMH level, homogeneity of follicular development and follicular size had a significant effect on embryo developmental competency, namely the top and good quality blastocyst rate.

In our study, selection of blastocysts for transfer was done according to the score obtained from the morphokinetic ratios published by Çetinkaya and colleagues and on the final morphology [29]. The ratios used in this algorithm do not favour the preferential selection of fast developing embryos, but require a synchrony in mitotic divisions. This is in line with the recent discussion that the speed of development needs to be within an optimal range, meaning that both too slow and too fast development will impact success rates [30]. The “quiet embryo hypothesis” postulates that early embryo viability is associated with a relatively stable-low metabolism [31]. The data on which this hypothesis was based were drawn from measurements on the depletion and appearance of amino acids from the culture medium. However, extending evidence on metabolic activity to the kinetics of pre-implantation embryo development, as recently reviewed by Leese and colleagues, may lead us to postulate that both too slow and too fast development result in lower success rates, due to non-optimal metabolic and/or genetic phenotype [30]. Therefore, intermediate kinetic ranges may be more physiological and may give better outcomes. Hence, as mentioned by these authors, a non-optimal metabolic status reflects the physiological and bioenergetic status of the embryo as a function of mitochondrial health. Because the mitochondrial pool, RNAs and proteins of an embryo were derived from the oocyte, the quality of follicular growth directly impacts embryo quality and viability. This is reflected in morphokinetic variables of early embryo development. This could also explain why embryos derived from small follicles achieved faster the blastocyst stage than those from large follicles, implying that in practice, the selection of embryos based on their rapidity may erroneously deselect embryos developing from large follicles which may have a higher implantation potential.

One other possible explanation for the different early developmental characteristics of embryos derived from small or large follicles could be a difference in cytoplasmic competence. Small follicles containing MII oocytes may have achieved nuclear competence but not necessarily cytoplasmic competence. Also, cytoplasmic maturation has recently been shown to be associated with mitochondrial distribution in mouse and human oocytes [15, 32].

The quiet embryo hypothesis also postulates that a low oxidative phosphorylation rate from the zygote to the morula stage limits reactive oxygen species production thus maximizing viability [33–35]. Oxidative stress can arise by the oxidative phosphorylation carried out by mitochondria and by the endoplasmic reticulum stress signalling [35]. We can speculate that the high bioenergetic level of small follicles leads to a precocious burnout reflected in morphokinetics, blastocyst and top and good quality blastocyst and implantation rates, thus inducing higher rates of developmental arrest and direct cleavages.

In conclusion, the highest rate of top and good quality blastocysts was achieved in embryos obtained from large follicles in homogenous cycles. A higher rate of direct cleavage and developmental arrest were observed in embryos obtained from small follicles. These findings can provide helpful information to both clinician and patient regarding the number of usable blastocysts likely to result in any particular cycle.

## Electronic supplementary material

Supplementary Table 1 (DOCX 16 kb)

## References

[1] Devreker F, Pogonici E, De Maertelaer V, Revelard P, Van den Bergh M, Englert Y (1999). Selection of good embryos for transfer depends on embryo cohort size: implications for the ‘mild ovarian stimulation’ debate. Hum Reprod.

[2] Opsahl MS, Blauer KL, Black SH, Lincoln SR, Thorsell L, Sherins RJ (2001). The number of embryos available for transfer predicts successful pregnancy outcome in women over 39 years with normal ovarian hormonal reserve testing. J Assist Reprod Genet.

[3] Fanchin R, Schonäuer LM, Cunha-Filho JS, Méndez Lozano DH, Frydman R (2005). Coordination of antral follicle growth: basis for innovative concepts of controlled ovarian hyperstimulation. Semin Reprod Med.

[4] Craft I, Gorgy A, Hill J, Menon D, Podsially B (1999). Will GnRH antagonists provide new hope for patients considered “difficult responders” to GnRH agonist protocol?. Hum Reprod.

[5] Wiesak K (2002). Role of LH in controlled ovarian stimulation. Reprod Biol.

[6] Nogueira D, Friedler S, Schachter M, Raziel A, Ron-El R, Smitz J (2006). Oocyte maturity and preimplantation development in relation to follicle diameter in gonadotropin-releasing hormone agonist or antagonist treatments. Fertil Steril.

[7] Depalo R, Lorusso F, Palmisano M, Bassi E, Totaro I, Vacca M (2009). Follicular growth and oocyte maturation in GnRH agonist and antagonist protocols for in vitro fertilisation and embryo transfer. Gynecol Endocrinol.

[8] Ectors FJ, Vanderzwalmen P, Van Hoeck J, Nijs M, Verhaegen G, Delvigne A, Schoysman R, Leroy F (1997). Relationship of human follicular diameter with oocyte fertilization and development after in-vitro fertilization or intracytoplasmic sperm injection. Hum Reprod.

[9] Salha O, Nugent D, Dada T, Kaufmann S, Levett S, Jenner L, Lui S, Sharma V (1998). The relationship between follicular fluid aspirate volume and oocyte maturity in in-vitro fertilization cycles. Hum Reprod.

[10] Rosen MP, Shen S, Dobson AT, Rinaudo PF, McCulloch CE, Cedars MI (2008). A quantitative assessment of follicle size on oocyte developmental competence. Fertil Steril.

[11] Farhi J, Orvieto R, Gavish O, Homburg R (2010). The association between follicular size on human chorionic gonadotropin day and pregnancy rate in clomiphene citrate treated polycystic ovary syndrome patients. Gynecol Endocrinol.

[12] Lee TF, Lee RK, Hwu YM, Chih YF, Tsai YC, Su JT (2010). Relationship of follicular size to the development of intracytoplasmic sperm injection-derived human embryos. Taiwan J Obstet Gynecol.

[13] Mehri S, Levi Setti PE, Greco K, Sakkas D, Martinez G, Patrizio P (2014). Correlation between follicular diameters and flushing versus no flushing on oocyte maturity, fertilization rate and embryo quality. J Assist Reprod Genet.

[14] Nivet AL, Léveillé MC, Leader A, Sirard MA (2016). Transcriptional characteristics of different sized follicles in relation to embryo transferability: potential role of hepatocyte growth factor signalling. Mol Hum Reprod.

[15] Sánchez F, Romero S, De Vos M, Verheyen G, Smitz J (2015). Human cumulus-enclosed germinal vesicle oocytes from early antral follicles reveal heterogeneous cellular and molecular features associated with in vitro maturation capacity. Hum Reprod.

[16] Van Blerkom J (2011). Mitochondrial function in the human oocyte and embryo and their role in developmental competence. Mitochondrion.

[17] Luciano AM, Lodde V, Franciosi F, Tessaro I, Corbani D, Modina S (2012). Large-scale chromatin morpho-functional changes during mammalian oocyte growth and differentiation. Eur J Histochem.

[18] Lemmen JG, Agerholm I, Ziebe S (2008). Kinetic markers of human embryo quality using time-lapse recordings of IVF/ICSI-fertilized oocytes. Reprod BioMed Online.

[19] Pribensky C, Matyas S, Kovacs P, Losonczi E, Zádori J, Vajta G (2010). Pregnancy achieved by transfer of a single blastocyst selected by time-lapse monitoring. Reprod BioMed Online.

[20] Wong CC, Loewke KE, Bossert NL, Behr B, De Jonge CJ, Baer TM (2010). Non-invasive imaging of human embryos before embryonic genome activation predicts development to blastocyst stage. Nat Biotechnol.

[21] Meseguer M, Herrero J, Tejera A, Hilligsøe KM, Ramsing NB, Remohí J (2011). The use of morphokinetics as a predictor of embryo implantation. Hum Reprod.

[22] Kirkegaard K, Agerholm IE, Ingerslev HJ (2012). Time-lapse monitoring as a tool for clinical embryo assessment. Hum Reprod.

[23] Bodri D, Sugimoto T, Serna JY, Kondo M, Kato R, Kawachiya S, Matsumoto T (2015). Influence of different oocyte insemination techniques on early and late morphokinetic parameters: retrospective analysis of 500 time-lapse monitored blastocysts. Fertil Steril.

[24] Bellver J, Mifsud A, Grau N, Privitera L, Meseguer M (2013). Similar morphokinetic patterns in embryos derived from obese and normoweight infertile women: a time-lapse study. Hum Reprod.

[25] Cruz M, Garrido N, Gadea B, Munoz M, Perez-Cano I, Meseguer M (2013). Oocyte insemination techniques are related to alterations of embryo developmental timing in an oocyte donation model. Reprod BioMed Online.

[26] Kirkegaard K, Hindkjaer JJ, Ingerslev HJ (2013). Effect of oxygen concentration on human embryo development evaluated by time-lapse monitoring. Fertil Steril.

[27] Muñoz M, Cruz M, Humaidan P, Garrido N, Pérez-Cano I, Meseguer M (2013). The type of GnRH analogue used during controlled ovarian stimulation influences early embryo developmental kinetics: a time-lapse study. Eur J Obstet Gynecol Reprod Biol.

[28] Wissing ML, Bjerge MR, Olesen AI, Hoest T, Mikkelsen AL (2014). Impact of PCOS on early embryo cleavage kinetics. Reprod BioMed Online.

[29] Cetinkaya M, Pirkevi C, Yelke H, Colakoglu YK, Atayurt Z, Kahraman S (2015). Relative kinetic expressions defining cleavage synchronicity are better predictors of blastocyst formation and quality than absolute time points. J Assist Reprod Genet.

[30] Leese HJ, Guerif F, Allgar V, Brison DR, Lundin K, Sturmey RG (2016). Biological optimization, the goldilocks principle, and how much is lagom in the preimplantation embryo. Mol Reprod Dev.

[31] Leese HJ (2002). Quiet please, do not disturb: a hypothesis of embryo metabolism and viability. BioEssays.

[32] Nagai S, Mabuchi T, Hirata S, Shoda T, Kasai T, Yokota S (2006). Correlation of abnormal mitochondrial distribution in mouse oocytes with reduced developmental competence. Tohoku J Exp Med.

[33] Baumann CG, Morris DG, Sreenan JM, Leese HJ (2007). The quiet embryo hypothesis: molecular characteristics favoring viability. Mol Reprod Dev.

[34] Landau G, Kodali VK, Malhotra JD, Kaufman RJ (2013). Detection of oxidative damage in response to protein misfolding in the endoplasmic reticulum. Methods Enzymol.

[35] Latham KE (2015). Endoplasmic reticulum stress signalling in mammalian oocytes and embryos: life in balance. Int Rev Cell Mol Biol.

