# Supplementary material for: The effect of follicle size and homogeneity of follicular development on the morphokinetics of human embryos
Source: J Assist Reprod Genet. 2017 May 4;34(7):895–903. doi: 10.1007/s10815-017-0935-1 (PMC5476546; doi:10.1007/s10815-017-0935-1)
Supplement: Supplementary file 1 — (DOCX 16 kb) [file 10815_2017_935_MOESM1_ESM.docx]

**Supplementary Table 1.** Clinical outcomes of patients

| **Patients (n:187)** | **n** | **%** |
| --- | --- | --- |
| **Clinical pregnancy** | 117 | 62.6 |
| **Ongoing pregnancy** | 101 | 54.0 |
| **Biochemical loss** | 8 | 6.4 |
| **Early clinical miscarriage** | 16 | 13.7 |
| **Patients with surplus frozen embryo(s)** | 124 | 66.3 |
| **Live birth*** | 93 | 50.8 |

* Live birth information could not be obtained from four couples.
